# Optimized Design of a Sub-Arc-Second Micro-Drive Rotary Mechanism Based on the Swarm Optimization Algorithm †

**DOI:** 10.3390/mi16101190

**Published:** 2025-10-21

**Authors:** Na Zhang, Dongmei Wang, Kai Li, Zhenyang Lv, Haochen Gui, Yizhi Yang, Manzhi Yang

**Affiliations:** 1 The Art College, Xi’an University of Science and Technology, Xi’an 710054, China; nazhang@xust.edu.cn (N.Z.); 13572817385@163.com (D.W.); 2 College of Mechanical Engineering, Xi’an University of Science and Technology, Xi’an 710054, China; lk149941@163.com; 3 School of Mechanical and Precision Instrument Engineering, Xi’an University of Technology, Xi’an 710000, China; 1220211007@stu.xaut.edu.cn; 4 Department of Mechanical Engineering, Shaanxi Tongchuan Industrial Technician College, Tongchuan 727000, China; ghc990925@163.com; 5 College of Humanities and Foreign Languages, Xi’an University of Science and Technology, Xi’an 710054, China; yangyizhi@xust.edu.cn

**Keywords:** micro-drive rotary system, structural optimization, sub-arc-second, drive performance, positioning performance, transformation performance

## Abstract

The optimization of the micro-motion rotary mechanism aims to obtain the maximum rotation angle in a certain space and increase the compensation range of the micro-motion mechanism. Aiming to address the disadvantages of a small movement stroke, low positioning accuracy, and limited research on the sub-arc-second level of precision micro-drive mechanism, a micro-drive mechanism was designed in this study and structural optimization was performed to obtain the maximum output angle. Additionally, the performance of the optimized mechanism was investigated. First, based on the principle of a flexure hinge guide and conversion, a micro-drive rotary mechanism that could transform the linear motion of piezoelectric ceramics into rotating motion accurately without parasitic motion and non-motion direction force was designed. Second, its structural optimization was achieved using the particle swarm optimization algorithm. Third, analyses of the drive performance and kinematics of the system were conducted. Finally, a performance test platform for the micro-drive rotary mechanism was built, its positioning performance and dynamic characteristics were verified experimentally, and the maximum rotary displacements and positioning error of the system were calculated. This research has certain reference value for studies of ultra-precision positioning.

## 1. Introduction

With the rapid development of high-precision positioning technology applied in the fields of medical devices, integrated circuit manufacturing, and high-precision machine tools, the positioning accuracy requirements of ultra-precise mechanical mechanisms have reached the nanometer level [1,2,3,4,5,6,7,8]. Current studies on precision micro-drive systems only focus on linear motion. Precision micro-drive systems with linear motion have reached positioning accuracy on the nanometer level and motion stroke on the micron level [9,10,11,12,13]. However, in fields such as laser measurement, semiconductors, and micro-robots, research on precision micro-drive systems for rotational motion is still insufficient [14,15,16].

A micro-drive rotary mechanism with a sub-arc-second level of rotation accuracy is expected to be widely used for ultra-high positioning accuracy. At the same time, the motion range of the micro-drive rotary mechanism will affect the compensation range of the macro-drive error in the macro/micro dual-drive rotary system. The larger the motion range of the micro-drive rotary mechanism, the larger the macro-drive error compensation of the micro-drive rotary system and the wider the application range of the macro/micro dual-drive rotary system [17,18]. Therefore, a sub-arc-second-grade precision micro-drive rotary mechanism with an optimized structure in a certain space was designed to achieve the goals of attaining the maximum output angle and completion of the related features. Studying sub-arc-second-grade micro-drive rotary mechanisms is important to promote ultra-precise micro-drive rotary technology research and development.

In recent years, many scholars have mainly focused on the mechanical structural design, structural optimization, dynamic analysis, and practical application of micro-drive systems [19,20,21,22]. Lin et al. [23] proposed a 12-DOF macro/micro dual-drive system composed of a 6-PSU mechanism and 6-SPS mechanism, which can achieve large-stroke motion and high-precision positioning. The experimental results show that the 12-DOF macro/micro dual-drive system has good performance, with a translational accuracy up to 15 μm, rotation accuracy up to 0.35′, and accuracy of the micromanipulator of more than ten nanometers. He Y X et al. [24] used a self-developed real-time embedded six-degree-of-freedom feedback system (6DFS) to develop a high-precision XY positioning platform that complies with the Abbe principle. This 6DFS includes three micro laser interferometers for XY positioning, as well as two dual-angle autocollimators for measuring the pitch, yaw, and roll angles. The experimental results show that the residual error of the three micro laser interferometers is ±10 nm; within the measurement range of ±30 arcseconds, the angular measurement resolution can reach 0.01 arcseconds; and after Brian error compensation, the *Z*-axis straightness error is reduced from −3 to 2 μm to ±100 nm. The performance of the XY platform was verified through contour testing, and the results show that its positioning error in the X and Y directions is ±10 nm. Zhu et al. [25] designed a novel compact flexure-based rotary micro positioning that is capable of a rotational angle of 1.58 µrad with the first natural frequency of 430 Hz, whilst the well-constrained maximum center shift along the X-and Y-axes are 0.29 mm and 0.12 mm, respectively, indicating a good decoupling capability. Furthermore, the compact size of 70 mm × 70 mm × 15 mm is beneficial for a limited working space. Zhang F et al. [26] proposed and designed a new type of six-degree-of-freedom micro-driving platform (6-DOF-MDS) that realizes micro-translation in the X, Y, and Z directions of the platform and micro-rotation around the three axes, enabling six-degree-of-freedom micro-feeding. Through a series of verification experiments, the stroke and resolution of this six-degree-of-freedom micro-driving platform were obtained: the displacements in the X, Y, and Z directions were 20.72 μm, 20.02 μm, and 37.60 μm, respectively, with a resolution better than 0.68 nm. Zhang Z et al. [27] successfully developed a new type of cross-cale micro–nano coordinate measuring machine (CSMN-CMM) compliant with the Abbe principle, which effectively resolved the contradiction between large-stroke motion and high-precision measurement in three-dimensional space. This motion system consists of a three-dimensional macro-motion platform and a six-degree-of-freedom (6-DOF) micro-motion platform, enabling both large-stroke and high-precision motion. The designed micro-motion platform has a linear displacement stroke exceeding 20.72 µm and a resolution better than 0.56 nm. The above research has been primarily centered on the motion and positioning performance of the systems. However, structural optimization studies aimed at the motion range of micro-rotary mechanisms are equally of great importance, and research in this regard remains inadequate to date.

The motion range of the micro-drive mechanism is an important performance indicator, and a micro-drive mechanism with a larger motion range has a wider application field. Therefore, optimizing and maximizing the motion range of the micro-drive rotary mechanism is important [28,29,30,31,32,33,34,35,36]. Therefore, this paper specifically focuses on investigating the optimization of the motion range of a sub-arcsecond-level micro-rotary mechanism.

The rest of this paper is organized as follows: Section 2 introduces the design of the micro-drive rotary mechanism. In Section 3, the structure of the micro-drive rotary mechanism is optimized. In Section 4, finite element analysis and experiments are performed on the micro-drive rotary mechanism. In Section 5, the performance of the micro-drive rotary mechanism is analyzed and discussed. The conclusions are presented in Section 6.

## 2. Design of the Micro-Drive Rotary Mechanism

In order to obtain sub-arc-second positioning accuracy, a micro-drive rotary mechanism was proposed that can accurately convert linear displacement into rotary displacement.

### 2.1. Working Principle of the Micro-Drive Rotary Mechanism

#### 2.1.1. Principle of Balanced Additional Force

**Figure 1:**
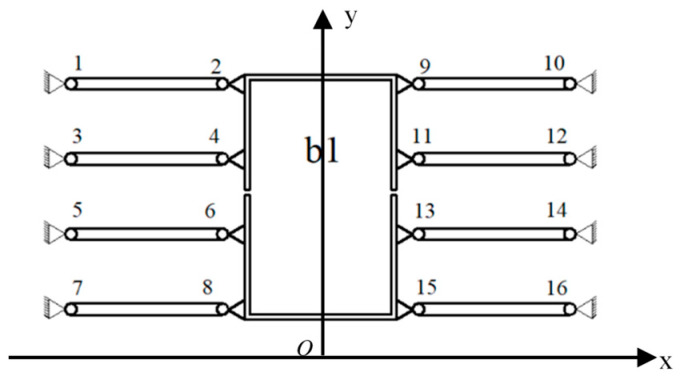
Principle of balancing additional forces.

The principle of balancing additional force is shown in Figure 1. The micro-driver is fixedly connected to b1, and the mechanism is symmetrically designed with eight flexible hinge components (flexible hinges 1–16). When the driver produces a drive displacement, the flexible hinge will be deformed. In addition to the force of the main direction of motion, forces in non-directions of motion are generated. At this time, the eight symmetrically distributed flexible hinge components have equal magnitude and opposite directions of force in the non-moving directions due to deformation, and the transverse forces from both directions can be balanced to achieve the additional force balance of the flexure hinge. Detailed principles can be found in reference [37] published by our research group.

#### 2.1.2. The Transmission Conversion Principle

**Figure 2:**
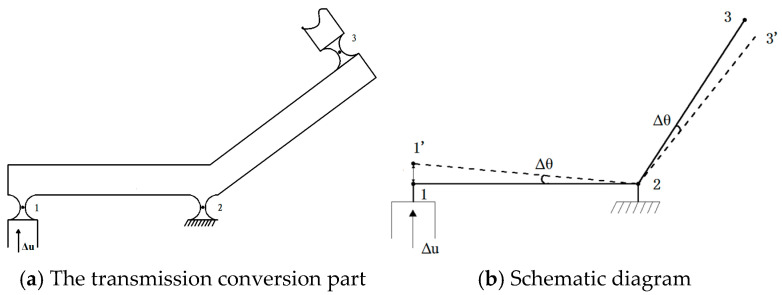
Schematic diagram of transmission conversion movement.

A schematic of the flexure hinge transmission conversion principle is shown in Figure 2, and is mainly composed of flexure hinge 1 connected to the input moving parts, flexure hinge 2 connected to the fixed base, flexure hinge 3 connected to the output moving parts, and rods 12 and 23, as shown in Figure 2a. Suppose that each section of the flexure hinge group is rigidly bonded and rods 12 and 23 are beam structures. A schematic of the flexure hinge transmission conversion movement is shown in the solid line in Figure 2b. When an instruction to move up, Δu, is given to the input moving part, because flexure hinge 1 is connected to the input moving part, flexure hinge 1 will also move Δu, where point 1′ is the position of point 1 after movement; because flexure hinge 2 is connected to the fixed base, rods 12 and 23 will rotate around point 2, and thus, the rotation angle Δθ will be generated. The schematic of the device after movement is shown as the dashed line in Figure 2b. Point 3′ is the position of point 3 after movement, and the rotation angle Δθ is generated, which is the output rotational displacement after transmission conversion. There are 26 flexible hinges in total in the micro-motion rotary mechanism. Since the mechanism is symmetrical, only its upper half needs to be analyzed. Among these hinges, flexible hinges 6–13 serve a guiding function but not a conversion function; hinges 4 and 5 are used to balance the lateral forces generated during the movement of hinges 1–3. Therefore, only hinges 1–3 are responsible for the absolute conversion of the angle magnitude, as shown in Figure 2.

### 2.2. Design of the Initial Mechanism

**Figure 3:**
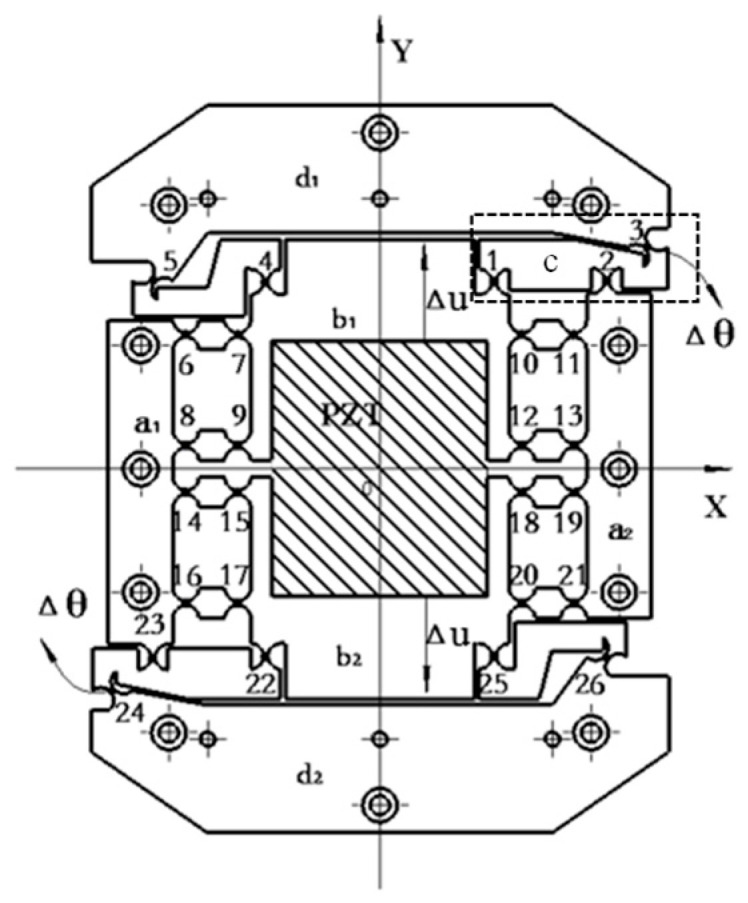
Diagram of the working principle of the micro-drive rotary mechanism.

A diagram of the working principle of the micro-drive rotary mechanism proposed in this paper is shown in Figure 3, which is mainly divided into four parts: connecting element a, input motion element b, flexure hinge c, and output motion element d. The micro-drive rotary mechanism has 26 flexure hinges (Nos. 1–26). The micro-drive rotary mechanism is symmetrical about the center of O point on the XY plane. When the PZT (piezoelectric actuator) inputs a micro displacement of 0.5 Δv along the positive direction of the *Y*-axis to the micro-drive rotary mechanism (when the output displacement of the PZT is Δv, it will input 0.5 Δv to the input motion element along the positive and negative directions of the *Y*-axis, respectively), the length $l_{12}$ of rod 12 becomes shorter, while the length $l_{23}$ of rod 23 becomes longer. Therefore, assuming that the distance $l_{1^{\prime }3^{\prime }}$ between point 1′ and point 3′ is equal to the distance $l_{13}$ between point 1 and point 3, the output angle Δθ of the output motion element is obtained when it receives the PZT input displacement Δu (Δu = 0.5 Δv).

Due to the symmetrical structure of the micro-drive rotary mechanism, the other part of the mechanism also produces the output angle Δθ under the action of the input dis-placement Δu of the PZT. Therefore, the micro-drive rotary mechanism achieves the precise conversion of Δu − Δθ. No lateral force or lateral displacement is generated during the movement, which ensures the safety of the PZT and the precise movement of the mechanism.

According to the micro-drive rotary mechanism shown in Figure 1, points 1–6 are defined as P_1_–P_6_, and the coordinates of P_1_ (x_1_, y_1_), P_2_ (x_2_, y_2_), P_3_ (x_3_, y_3_), P_4_ (x_4_, y_4_), P_5_ (x_5_, y_5_), and P_6_ (x_6_, y_6_) of the flexure hinges responsible for transmission transformation can be obtained, as shown in Table 1. R_2_, R_3_, and R_5_ are the distances from points 2, 3 and 5 to the origin O. θ_2_, θ_3_, and θ_5_ are the angles between R_2_, R_3_, and R_5_ and the *X*-axis. In the motion analysis, the connecting element a, the input motion element b, and the output motion element d are regarded as rigid bodies, and the 12 connecting rods, such as *l*_123_, *l*_45_ and *l*_67_, are regarded as rigid rods; 26 hinges such as 1, 2, 3, 4 flexure hinges. According to the motion performance of the micro-drive rotary mechanism, the relation between the straight line input displacement Δu and the rotation output angle Δθ can be established by transforming the equation as follows:

If rod 13 is regarded as a rigid rod, then

(1) $$l_{13\;}={\;l}_{1^{\prime }3^{\prime }}$$

The following can be obtained from the geometric relationship of rod 13 in Figure 1: $l_{13}=\sqrt{{\left(R_{3}\cos\theta -x_{1}\right)}^{2}+{\left(R_{3}\sin\theta -y_{1}\right)}^{2}}$, $\;{\;l}_{1^{\prime }3^{\prime }}=\sqrt{{\left[R_{3}\cos\left(\theta +\Delta \theta \right)-x_{1}\right]}^{2}+{\left[R_{3}\sin\left(\theta +\Delta \theta \right)-y_{1}-\Delta u\right]}^{2}}\;$. Substituting this equation into Equation (1) obtains

(2) $$(2y_{1}R_{3}+2\Delta uR_{3})\sin\left(\theta +\Delta \theta \right)+2x_{1}R_{3}\cos\left(\theta +\Delta \theta \right)={\Delta u}^{2}+2\Delta uy_{1}+2x_{1}R_{3}\cos\theta +2y_{1}R_{3}\sin\theta$$

$k_{1}$, $k_{2}$ and $k_{3}$ are constants. Let $k_{1}=2y_{1}R_{3}+2\Delta uR_{3}$, $k_{2}=2x_{1}R_{3}$, and $k_{3}={\Delta u}^{2}+2\Delta uy_{1}+2x_{1}R_{3}\cos\theta +2y_{1}R_{3}\sin\theta$; Equation (2) can be changed to

(3) $$k_{1}\sin\left(\theta +\Delta \theta \right)+k_{2}\cos\left(\theta +\Delta \theta \right)={\;k}_{3}$$

After arranging Equation (3), the following equation can be obtained:

(4) $$\Delta \theta \;=\mathrm{atan}2\left(k_{2^{\prime }}-k_{1}\right)-\mathrm{atan}2(k_{3^{\prime }}\pm \sqrt{k_{1}^{2}{+k}_{2}^{2}{-k}_{3}^{2}})-\theta$$

As shown in Equation (4), when the flexure hinge responsible for the transformation motion of the micro-drive rotary mechanism is known, the equation becomes a functional expression of the rotational output angle Δθ that is only related to the linear input displacement Δu.

## 3. Optimizing the Structure of the Micro-Drive Rotary Mechanism

To obtain the maximum output angle of the micro-drive rotary mechanism, the structure of the micro-drive rotary mechanism was optimized. After comparing and analyzing particle swarm optimization, ant colony optimization, and the genetic algorithm, the PSO algorithm was selected for this study. The problem addressed in this paper is a typical complex optimization problem that exhibits three key characteristics: its objective function tends to be non-convex and multimodal; the problem model lacks differentiability, rendering gradient-based optimization methods inapplicable; and the cost of function evaluation is relatively high. To tackle these challenges, the PSO algorithm offers distinct advantages: it excels at addressing multimodality and preventing premature convergence, simultaneously handles non-differentiable problems effectively, and ultimately achieves high optimization efficiency and stable convergence. Therefore, the PSO algorithm was chosen for this study.

### 3.1. Optimized Mathematical Model

In this algorithm, the number of individuals, location range, and movement speed of the population need to be given first. The fitness of each particle is calculated subsequently. The current fitness of each particle is compared with its previously discovered optimal solution pb and its previously discovered optimal solution g_b_ to select the optimal particle in this iteration. Since the population searches for the optimal solution within the position range, the velocity and position of the particle are updated using Equations (5) and (6) [38,39,40]:

(5) $$v_{i}^{k+1}=\omega \;\times {\;v}_{i}^{k}\;+\;c_{1}\times \;{\mathrm{rand}}_{1}\times \left({p_{b}}_{i}-x_{i}^{k}\right)+\;c_{2}\times {\;\mathrm{rand}}_{2\;}\times \;\left({g_{b}}_{i}-x_{i}^{k}\right)$$

(6) $$x_{i}^{k+1}=x_{i}^{k}+v_{i}^{k+1}$$

In Equations (5) and (6), i = 1, 2, …, M. M is the total number of particles in the group; $v_{i}^{k}$ is the velocity of the ith particle at the Kth iteration; $x_{i}^{k}$ is the position of the ith particle in the Kth iteration; ω is the inertia weight factor, usually 0.5; rand_1_ and rand_2_ are random numbers between (0, 1); c_1_ and c_2_ are learning factors, where c_1_ = c_2_ = 2; the population size is 100; and the number of iterations is 500.

### 3.2. Determination of the Design Variables

Using the structure shown in Figure 1 showing the micro-drive rotary mechanism about the origin O center of symmetry, only the first half of micro-drive rotary mechanism needs to be analyzed. The roles of flexure hinges 4 and 5 are to balance the lateral force and moment produced when the linear displacement of components 1, 2, and 3 are converted to rotary displacement. Therefore, the optimization goal is transformed to achieving the coordinate values of the optimized hinges 1, 2, and 3. Therefore, the design variables x_1_, x_2_, and x_3_ are the abscissa values (mm) and x_4_, x_5_, and x_6_ are the ordinate values (mm) of flexure hinges 1, 2 and 3, respectively.

### 3.3. Establishment of the Objective Function

According to Equation (4) for the kinematic analysis of the micro-drive rotary mechanism, the constant 7.5 μm (which is the maximum output value of the PZT) is substituted into the input value Δu in $k_{1}$ and $k_{3}$, $\sqrt{x_{3}^{2}{+x}_{6}^{2}}$ is substituted into the distance R_3_, $\arcsin(\frac{x_{6}}{\sqrt{x_{3}^{2}{+x}_{6}^{2}}})$ is substituted into $k_{3}$, and the angle θ is substituted in Equation (4). In this case, Equations (7)–(9) are obtained, and Equation (4) becomes a function whose output values Δθ are dependent only on the coordinates of hinges 1, 2, and 3.

During structural optimization, the objective is to obtain the maximum value of the output rotary angle Δθ. Therefore, the objective function maxf(x) = Δθ can be obtained by substituting it into Equation (10):

(7) $$k_{1}\;=\;2\;\times \;x_{4}\times \sqrt{x_{3}^{2}{\;+\;x}_{6}^{2}}\;+\;2\;\times \;0.0075\;\times \;\sqrt{x_{3}^{2}\;{+\;x}_{6}^{2}}$$

(8) $$k_{2}=2\;\times \;x_{1}\;\times \;\sqrt{x_{3}^{2}{+x}_{6}^{2}}\;$$

(9) $$k_{3}={\;0.0075}^{2}+2\;\times \;0.0075\;\times {\;x}_{4}+2\;\times \;x_{1}\;\times \;\sqrt{x_{3}^{2}{+x}_{6}^{2}}\;\times \;\cos(\arcsin\frac{x_{6}}{\sqrt{x_{3}^{2}{+x}_{6}^{2}}})+\;\;2\;\times \;x_{4}\;\times \;\sqrt{x_{3}^{2}{+x}_{6}^{2}}\;\times \;\sin(\arcsin\frac{x_{6}}{\sqrt{x_{3}^{2}{+x}_{6}^{2}}})\;$$

(10) $$\Delta \theta =\arctan(-\frac{k_{2}}{k_{1}})+\arctan(\frac{k_{3}}{\sqrt{k_{1}^{2}{+k}_{2}^{2}{-k}_{3}^{2}}})-\arcsin(\frac{x_{6}}{\sqrt{x_{3}^{2}{+x}_{6}^{2}}})\;$$

This study, which targets highly nonlinear systems, adopts the dominant term decomposition method for a structured qualitative analysis: $k_{1}$ is most sensitive to $x_{4}$, and its sensitivity to $x_{3}$ and $x_{6}$ depends on their relative magnitudes. $k_{2}$ is a linear function that is most sensitive to $x_{1}$, with its sensitivity to $x_{3}$ and $x_{6}$ depending on their relative magnitudes. $k_{3}$ is a linear function, exhibiting the highest sensitivity to $x_{1}$ and $x_{4}$ (direct product term) and lower sensitivity to $x_{3}$ and $x_{6}$. The sensitivity of Δθ is highly nonlinear and dependent on the operating point. This conclusion has met the requirements of subsequent optimization design. Future research will introduce an adaptive sensitivity threshold in global optimization to further improve accuracy. According to Equation (11), when the linear input displacement Δu is constant, the output rotary angle Δθ of the micro-drive rotary mechanism is only related to the coordinates of the flexure hinges 1, 2, and 3; thus, the optimal solution of the output rotary angle Δθ of the micro-drive rotary mechanism can be found by optimizing the coordinates of the flexure hinges 1, 2, and 3.

(11) $$\max\;f\;\left(x\right)=\arctan(-\frac{k_{2}}{k_{1}})+\arctan(\frac{k_{3}}{\sqrt{k_{1}^{2}{+k}_{2}^{2}{-k}_{3}^{2}}})-\arcsin(\frac{x_{6}}{\sqrt{x_{3}^{2}{+x}_{6}^{2}}})\;$$

### 3.4. Establishment of Constraints

#### 3.4.1. The Position Condition of the X-Direction Point

Because the structure is optimized on the original size of the micro-drive rotary mechanism and the original mechanism is symmetric about the origin O, the x-point positions of hinges 1, 2, and 3 are between 0 and 67 (mm). According to experience, in order to prevent rod *l*_23_ from being too small to bend or break, *l*_23_ ≥ 8 is required. The following conditions must be met:

(12) $$0\;<{\;x}_{1}\;<\;42\;$$

(13) $$x_{1}\;<{\;x}_{2}\;<\;59$$

(14) $$x_{2\;}<\;x_{3}\;<\;67\;$$

In the above equations, ${x_{1},x}_{2}{,and\;x}_{3}$ need to be rounded.

#### 3.4.2. The Position Condition of the Y-Direction Point

Because the structure is optimized on the original size of the micro-drive rotary mechanism and the original mechanism is symmetric about the origin O, the space for placing the PZT should be 70 mm in the Y direction in the middle of the micro-drive rotary mechanism (half is 35 mm and the radius of the semi-arc of the flexure hinge is 3 mm; thus, it is 38 mm); hinges 1, 2, and 3 are between 38 and 85.5 mm in the Y direction. According to experience, *l*_23_ ≥ 8 is required to prevent the input displacement from being converted into output displacement that is too small. The following conditions must be met:

(15) $$38\;<\;x_{4\;}<{\;x}_{5}$$

(16) $$x_{4}\;<{\;x}_{5}\;<\;77.5\;$$

(17) $$x_{5}\;<{\;x}_{6\;}<\;85.5\;$$

In the above equation, $x_{4},x_{5}{,and\;x}_{6}$ need to be rounded.

#### 3.4.3. Collinear Conditions

To ensure that the structural optimization will not affect the rotary motion and will output the maximum rotary angle, the origin O and hinges 2 and 3 should be collinear; therefore, the following conditions should be met:

(18) $$\frac{x_{5}}{x_{2}}\;=\;\frac{x_{6}}{x_{3}}\;$$

(19) $$\sqrt{x_{3}^{2}{+x}_{6}^{2}}-\sqrt{x_{2}^{2}{+x}_{5\;}^{2}}\geq \;8$$

In the above equations, $x_{2}{,\;x}_{3},{\;x}_{5}{,\;\mathrm{and}\;x}_{6}$ need to be rounded.

### 3.5. Optimized Micro-Drive Rotary Mechanism

**Figure 4:**
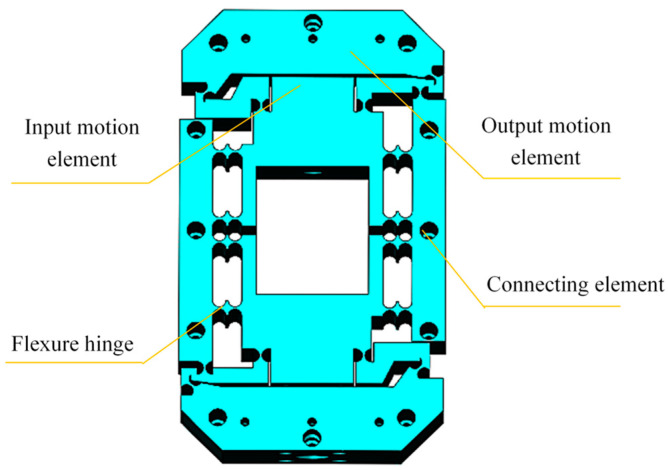
Diagram showing a model of the optimized structure of the micro-drive rotary mechanism.

The issue was solved using MATLAB 2022a on a Mechrevo (MECHREVO, Xi’an, China) computer (equipped with an Intel(R) Core(TM) i7-10750H CPU @ 2.60GHz), and the new position coordinates of the flexure hinges of the micro-drive rotary mechanism were obtained: flexure hinge 1 (23, 58), flexure hinge 2 (48, 64), and flexure hinge 3 (54, 72). The theoretical calculations of the micro-drive rotary mechanism optimized using PSO and those before optimization are listed in Table 2 for comparison, and the results show that the theoretical travel increases by 58.2% (the average rate of increase). The optimized structure model of the micro-drive rotary mechanism is shown in Figure 4.

## 4. Analysis and Experiment

### 4.1. Kinematic Analysis

**Figure 5:**
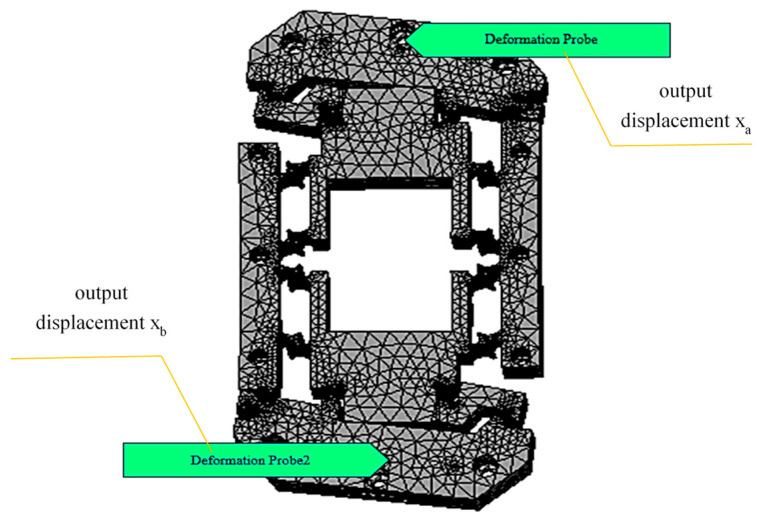
Deformation of the micro-motion rotary mechanism when the input quantity is 1.01 μm.

The finite element results for the output displacement of the micro-drive rotary mechanism are shown in Figure 5. By changing the input displacement, the micro-drive rotary mechanism can output different size displacements. The positive and negative displacements of *X*-axis at point (0, 90, 50) and point (0, −90, 50) on the model are measured successively. When the *Y*-axis exerts positive and negative directional displacements, Δu is 1.01 μm and the positive and negative displacements of the *X*-axis measured by the probe at point (0, 90, 50) and point (0, −90, 50) calculated using the static analysis are 0.846 μm and −0.773 μm, respectively; therefore, Δθ = arctan(0.00162/180) = 1.86″. The deformation is shown in Figure 5. When the applied displacement Δv is 2.02 μm (Δu = 1.01 μm), the output angle Δθ of the micro-drive rotary mechanism is 1.86″. Similarly, the output angle Δθ of the micro-drive rotary mechanism corresponding to different Δu values is obtained, as shown in Table 2.

In the finite element analysis, the output angle of the optimized micro-drive rotary mechanism increases by 53.4% (the average rate of increase) compared with that of the pre-optimized micro-drive rotary mechanism. The error of the theoretical calculation and finite element analysis of the optimized micro-drive rotary mechanism is 8.12% (maximum error), which meets the design requirements of the micro-drive rotary mechanism. This error arises from two key aspects. First, the theoretical calculation is overly idealized and fails to account for practical factors such as stiffness. Second, the finite element calculation introduces errors, which stem from issues like modeling, mesh generation, and boundary conditions.

### 4.2. Experiment Assessing the Drive Characteristics of Mechanism

The accuracy of the output angle of the mechanism will be affected by the drive characteristics of the mechanism. Piezoelectric ceramics have characteristics such as hysteresis, creep, and nonlinearity. However, the PZT of model P235.1s from PI Corporation (Auburn, MA, USA) that we adopted is equipped with built-in feedback components and control algorithms, which have minimized the hysteresis effect to the greatest extent. Its response frequency and resolution are 300 Hz and 0.3 nm, respectively. The drive characteristics of the PZT used by the mechanism were tested.

**Figure 6:**
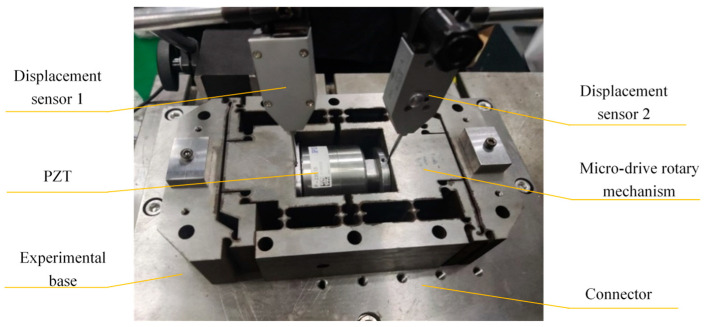
The experimental setup for assessing drive characteristics.

The setup of the experiment assessing the drive characteristics is shown in Figure 6. To minimize the impact of temperature, the experiment was conducted under constant temperature conditions (20–22 °C, with fluctuations less than 2 °C). The experimental device is mainly composed of the experimental base, P235.1s PZT, micro-drive rotary mechanism, connector, two-side displacement sensors (Zhongyuan Measurement DGC-6PG/A: measurement range ±0.3 mm, total stroke 1–1.5 mm, linear error ±0.5%, and repeatability error 0.05 μm), data recorder, piezoelectric ceramic controller, etc. By changing the driving voltage U, the displacement variation of the micro-drive rotary mechanism along the *Y*-axis is measured by the sided displacement sensors 1 and 2, which are δy_1_ and δy_2_, respectively. Because the PZT is driven symmetrically in the micro-drive rotary mechanism, the maximum displacement of unilateral drive is 7.5 μm. The driving displacement Δv of PZT can be expressed as follows:

(20) $$\Delta v\;=\;\left|{\delta y}_{1}\right|\;+\;\left|{\delta y}_{2}\right|$$

**Figure 7:**
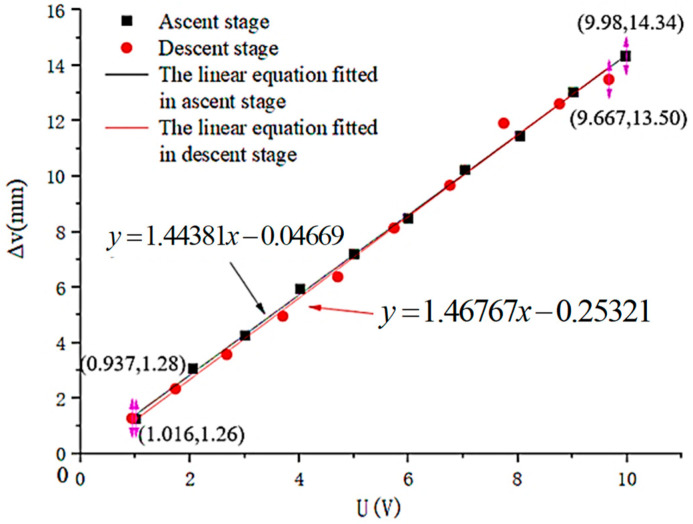
Assessment of drive performance.

The experiment was conducted five times, and the average value was taken as the final experimental data result. Then, linear programming was performed on the final experimental data. The drive performance of the micro-drive rotary mechanism was tested in the ascending stage (when the driving voltage U increases) and the descending stage (when the driving voltage U decreases). The experimental results are shown in Figure 7.

The fit of the linear equation between the drive voltage U and the linear motion displacement Δv of the mechanism in the ascending stage is as follows:

(21) Δv = 1.44381U − 0.04669

The linearity of the linear equation is 0.9991.

The fit of the linear equation between the drive voltage U and the linear motion displacement Δv of the mechanism in the descending stage is as follows:

(22) Δv = 1.46767U − 0.25321

The linearity of the linear equation is 0.9936.

The drive performance equation can be analyzed from Equations (21) and (22) as follows:

(23) $$\Delta v\;=\;\left\{\begin{array}{c} 1.44381U\;-\;0.04669\left(\mathrm{Ascent}\;\mathrm{stage}\right)\\ \;1.46767U\;-\;0.25321\left(Descentstage\right) \end{array}\right.$$

The minimum linearity of the linear equation is 0.9936.

The drive error of the mechanism δ_1_ can be calculated from the slopes of the ascending and descending stages using Equation (23), as follows:

(24) $$\delta_{1}\;=\;\frac{A_{2}-A_{1}}{A_{2}}\;\times \;100\%\;=\;1.63\%$$

where A_1_ and A_2_ are the slopes of the ascending and descending stages in Equation (24).

Therefore, drive performance was analyzed using Equation (24). The minimum linearity of the linear of the equation was 0.9936, and the drive error of the mechanism δ_1_ was 1.63%.

### 4.3. Experiment Assessing Positioning Performance

The relation between the linear displacement Δv and output angle Δθ (Δv − Δθ) directly affects the kinematic performance of the mechanism. Assessments of the kinematic performance of the mechanism must be conducted.

**Figure 8:**
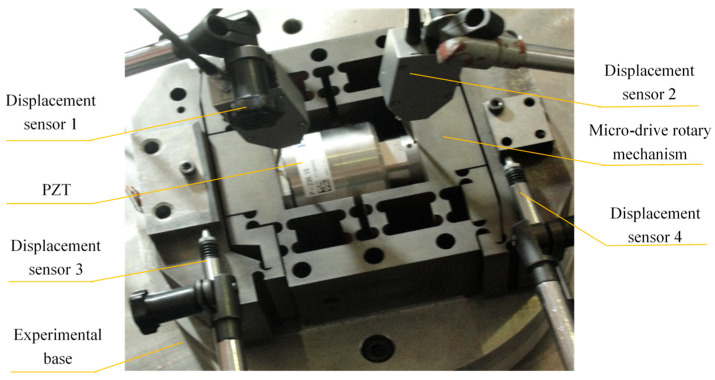
Diagram of the experimental setup for assessing the kinematic performance of the mechanism.

To accurately analyze the positioning performance during the experiment, different driving voltages, U, are input to the PZT. To minimize the impact of temperature, the experiment is conducted under constant temperature conditions (20–22 °C, with fluctuations below 2 °C). The positioning performance experiment is shown in Figure 8. The experimental device is mainly composed of the experimental base, P235.1s PZT, micro-drive rotary mechanism, connectors, two-side displacement sensors, two straight displacement sensors DGC-8ZG/C(Zhongyuan Measurement, Xi’an, China): measurement range ±0.6 mm, total stroke 3 mm, linear error ± 0.5%, and repeatability error 0.03 μm, a data recorder, and a piezoelectric ceramic controller. During the experiment, the displacements δy_1_ and δy_2_ in the *Y*-axis direction were measured by displacement sensors 1 and 2, and the linear input displacement Δu of the micro-drive rotary mechanism was obtained. The output angle Δθ of the micro-drive rotary mechanism could be indirectly determined by measuring the displacement δx_1_ and δx_2_ in the *X*-axis direction using displacement sensors 3 and 4. This experimental method is the same as that described in Section 4.2.

According to the displacement changes measured by the above four displacement sensors, the displacement values Δu and Δθ of the PZT input and output of the micro-drive rotary mechanism can be calculated from Equations (25) and (26), respectively:

(25) $$\Delta v\;=\;\left|{\delta y}_{1}\right|\;+\;\left|{\delta y}_{2}\right|$$

(26) $$\Delta \theta =\tan^{-1}\left(\frac{\left|{\delta x}_{1}\right|\left|{\delta x}_{2}\right|}{l}\right)=\tan^{-1}\left(\frac{\left|{\delta x}_{1}\right|\left|{\delta x}_{2}\right|}{180}\right)$$

**Figure 9:**
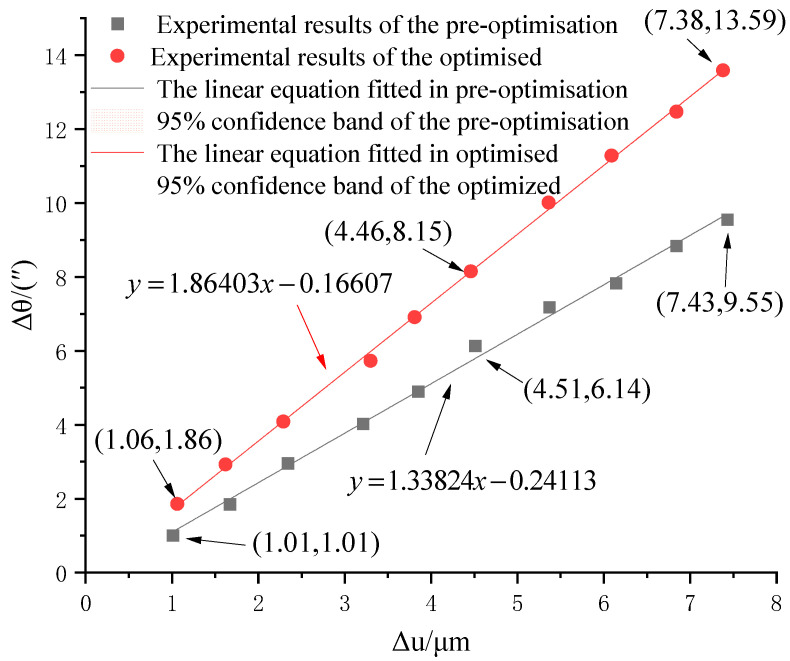
Experimental results for kinematic performance.

Different linear input displacement values, Δu, can be obtained using Equation (25), and then the rotary angle Δθ of the corresponding micro-drive rotary mechanism can be obtained using Equation (26). The experimental results of the micro-drive rotary mechanism before and after optimization are shown in Table 3 and Figure 9.

Figure 9 incorporates 95% confidence intervals, which were calculated using the mean values derived from five independent experimental replicates. Given the small sample size (*n* = 5), a t-distribution with 4 (*n* − 1) degrees of freedom was employed to compute these intervals.

The linear equation fitted to the kinematic performance of the pre-optimized micro-drive rotary mechanism is

(27) Δθ=1.33824Δu−0.24113

The linearity of the linear equation is 0.9963.

The linear equation fitted to the kinematic performance of the optimized micro-drive rotary mechanism is

(28) Δθ=1.86403Δu− 0.16607

The linearity of the linear equation is 0.9990.

## 5. Performance Analysis and Discussion

### 5.1. Analysis of the Conversion Characteristics

**Figure 10:**
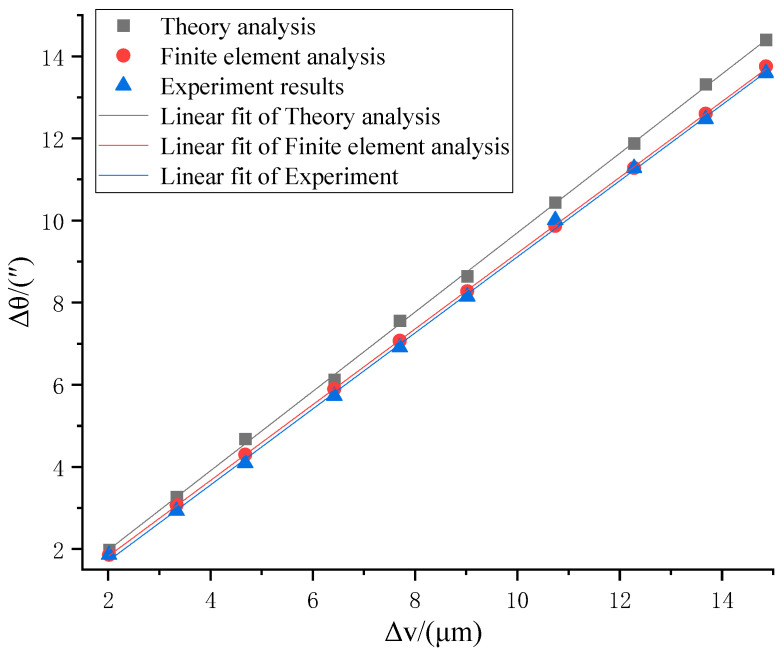
Linear fits of the conversion characteristics.

To analyze the linear input displacement Δv of the PZT and the rotary angle Δθ of the optimized micro-drive rotary mechanism, linear fits were obtained using three methods, theory analysis, finite element analysis, and the experimental results, and the linear fits are shown in Figure 10.

The linear equation fitted to the theory analysis of kinematic performance is

(29) Δθ=0.96641Δv+0.03965

The linearity of the linear equation is 0.9996.

The linear equation fitted to the finite element analysis of kinematic performance is

(30) Δθ=0.92301Δv − 0.02354

The linearity of the linear equation is 0.9999.

The linear equation fitted to the experimental results for kinematic performance is

(31) Δθ=0.93202Δv − 0.16607

The linearity of the linear equation is 0.9990.

The linearity of the fitted equations of the optimized micro-drive rotary mechanism from the theory analysis, finite element analysis, and experimental results is at least 0.9990, indicating good linearity. The coefficients of Δu in Equations (29)–(31) can be averaged for practical application, and the kinematic performance conversion characteristics of the mechanism can be described using Equation (32):

(32) Δθ = 0.9405Δv

The resolution of the optimized micro-drive rotary mechanism can be derived from the resolution of 0.0003 mm for the P235.1s PZT combined with Equation (32) to be “0.0003 × 0.9405 = 0.00028”. Therefore, the optimized resolution of the micro-drive rotary mechanism is “0.00028”.

The error between the experimental results and the theoretical calculation of the optimized micro-drive rotary mechanism is 7.13%, while the error from the finite element analysis is 1.88%, and thus the maximum error of the mechanism’s positioning performance conversion δ_2_ is 7.13%.

### 5.2. Analysis of the Positioning Performance

The positioning performance of the mechanism is determined by studying the conversion relationship between the driving voltage U and the output angle Δθ (i.e., the function between U and Δθ) during the movement of the optimized micro-drive rotary mechanism.

Based on Equation (23) for the drive performance and Equation (32) for the kinematic conversion characteristics, the positioning performance equation for the optimized micro-drive rotary mechanism can be obtained as follows:

(33) $$\Delta \theta \;=\;\left\{\begin{array}{c} 1.5352U\;-\;0.0496\left(\mathrm{Ascent}\;\mathrm{stage}\right)\\ \;1.5605U\;-\;0.2692\left(Descentstage\right) \end{array}\right.$$

The linearity of the ascending and descending stages of Equation (33) is 0.9991 and 0.9936, respectively, from the analysis, and the minimum linearity of Equation (33) is 0.9936.

If the positioning error of the mechanism is δ, then

(34) $$\delta =1-\left(1-\delta_{1}\right)\left(1-\delta_{2}\right)$$

where δ_1_ is the optimized drive error of the micro-drive rotary mechanism, with δ_1_ = 1.63%, and δ_2_ is the optimized maximum error of the kinematic conversion of the micro-drive rotary mechanism, with δ_2_ = 7.13%. Therefore, the positioning error δ of the optimized micro-drive rotary mechanism is 8.64%.

In practice, the mid-point voltage is generally used as the zero voltage, and the maximum drive voltage of the PZT U_max_ = 10V. The insertion of the PZT drive voltage U and the optimization of the positioning error of the micro-drive rotary mechanism δ = 8.64% into Equation (33) allows the maximum positioning performance motion displacement matrix A for the ascending and descending stages of the optimized micro-rotary mechanism to be obtained as follows:

(35) $$A=\left[\begin{array}{cc} {\Delta \theta }_{1\max} & e_{1\max}\\ {\Delta \theta }_{2\max} & e_{2\max} \end{array}\right]=\left[\begin{array}{cc} 15.30^{\prime \prime } & 0.66^{\prime \prime }\\ 15.34^{\prime \prime } & 0.67^{\prime \prime } \end{array}\right]$$

In Equation (35), Δθ_1max_ and Δθ_2max_ are the maximum output angles of the optimized micro-drive rotary mechanism in the ascending and descending stages, and e_1max_ and e_2max_ are the maximum positioning error values of the optimized micro-drive rotary mechanism in the ascending and descending stages.

**Figure 11:**
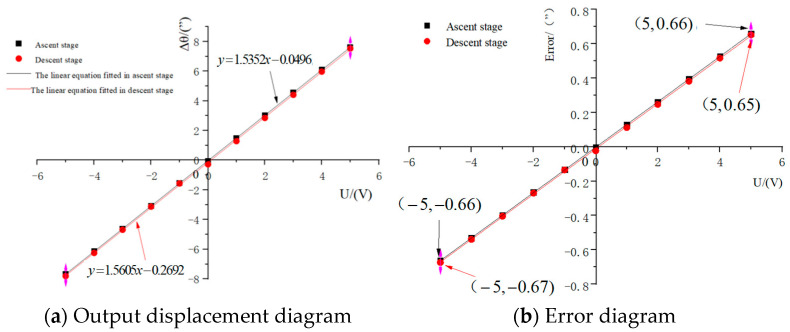
Maximum kinematic displacement of the optimized micro-drive rotary mechanism.

The maximum kinematic displacements and errors of the optimized micro-drive rotary mechanism in the ascending and descending stages are shown in Figure 11a and Figure 11b, respectively.

Based on the results of the analysis of the positioning performance of the optimized micro-drive rotary mechanism, the following conclusions can be drawn:

(1) The optimized mechanism has excellent drive performance and a minimum linearity of 0.999.

(2) The maximum positioning error value of the optimized micro-drive rotary mechanism is 0.67″, thus providing a sub-arc-second rotary angle.

(3) The maximum output rotary angle of the optimized micro-drive rotary mechanism is 15.34″ and the positioning error is 8.64%; therefore, the positioning range of the optimized micro-drive rotary mechanism is approximately 0″–15.34″, and the sub-arc-second level was achieved.

### 5.3. Discussion

The maximum output angle of the mechanism designed in this study is compared with that of other micro-motion mechanisms discussed in the literature in Table 4. The maximum positioning error values of the mechanism are compared with those of other micro-motion mechanisms addressed in the literature in Table 5.

As can be observed in Table 4 and Table 5, the mechanism designed in this study demonstrates a notable increase in the maximum output angle, and its maximum positioning error value is significantly smaller than those of other mechanisms, indicating a substantial enhancement in motion accuracy.

In comparison to the original design, the mechanism described in this paper exhibits a significantly expanded slewing range (from 0″–8.91″ to 0″–15.34″). As can be seen in Table 5, the maximum positioning error of this mechanism is closest to that of Reference [16], while multiple gaps are observed when compared with other references in the table. The calculation method for the maximum positioning error in this paper is the same as that in Reference [16]. Regardless of whether the calculation methods for positioning errors are the same as those of the other four references, it can be concluded, through comparison, that the maximum positioning error of the mechanism in this paper is the smallest at 0.67″.

Meanwhile, this study takes the output rotary displacement Δθ as the sole optimization objective and utilizes optimization algorithms to determine the structural parameters required to achieve the maximum motion output. However, other performance indicators of the micro-motion rotary mechanism, such as rigidity and stability, are also crucial factors that demand consideration in the optimization of such micro-motion mechanisms. Specifically, rigidity affects the deformation degree of the mechanism at non-operating positions, while stability influences the overall displacement performance of the mechanism; both of these factors directly impact the motion accuracy of the system. Therefore, in-depth research into these aspects is essential. Subsequent research efforts should focus on ensuring the optimal performance and accuracy of precision calculations related to the motion characteristics of the optimized micro-motion rotary mechanism.

## 6. Conclusions

The main research purpose of this study is to design an optimization algorithm for a micro-motion slewing mechanism within certain size and structural ranges to obtain a larger rotational output displacement Δθ through the mechanism conversion function at the same linear input displacement Δu. This enables the micro-motion mechanism to have a larger motion stroke, thereby achieving better performance in the compensation range of macro-micro drive systems or in the independent motion of micro-motion mechanisms. The results show that the positioning accuracy of the optimized micro-drive rotary mechanism can reach the sub-arc-second level and the positioning performance is favorable.

(1) A micro-drive rotary mechanism capable of converting linear displacement into rotary displacement free from non-motion directional forces and displacements was developed.

(2) PSO was used to optimize the structure of the micro-drive rotary mechanism and increase the output angle of the mechanism.

(3) The experimental results showed that the positioning range of the optimized micro-drive rotary mechanism was approximately 0″–15.34″, the maximum positioning error value of the optimized micro-drive rotary mechanism was 0.67″, and the sub-arc-second level was achieved.

(4) In theoretical calculations, the motion range of the optimized micro-drive rotary mechanism increased by 58.2% compared with that before optimization, and the errors of the finite element analysis and experimental results were 8.12% and 7.13%, respectively. The optimized mechanism has a wider compensation range and can be used to compensate for macro-motion systems with lower motion accuracy.

This study has an important reference value for sub-arc-second positioning precision of the rotary angle and the optimization of micro-drive rotary mechanism structure, and will be helpful to promote the development of high-precision micro-drive rotary technology.

## Figures and Tables

**Table 1:** The initial conditional parameters.

Parameter	x_1_	y_1_	x_4_	y_4_	R_2_	θ_2_	R_3_	θ_3_	R_5_	θ_5_
value	26.5	44.0	−26.5	44.0	68.8	40.0	81.0	40.0	68.8	140.0

**Table 2:** The relationship between Δu and Δθ before and after optimization.

Input Values Δu/μm	Theoretical Δθ/(″)		Rate of Increase/(%)	Finite Element Analysis Δθ/(″)		Rate of Increase/(%)	The Optimized Error/(%) (θ_1_ − θ_2_)/θ_1_
	Before Optimization	After Optimization θ_1_		Before Optimization	After Optimization θ_2_		
1.01	1.24	1.98	0.597	1.21	1.86	0.537	0.0606
1.67	2.06	3.27	0.587	2.00	3.07	0.535	0.0612
2.34	2.88	4.68	0.625	2.81	4.30	0.530	0.0812
3.21	3.95	6.12	0.549	3.85	5.90	0.532	0.0359
3.85	4.74	7.56	0.595	4.62	7.07	0.530	0.0648
4.51	5.55	8.64	0.557	5.41	8.28	0.530	0.0417
5.37	6.61	10.44	0.579	6.44	9.87	0.533	0.0546
6.14	7.56	11.88	0.571	7.37	11.28	0.531	0.0505
6.84	8.42	13.32	0.582	8.21	12.60	0.535	0.0541
7.43	9.15	14.40	0.574	8.91	13.75	0.543	0.0451

**Table 3:** Comparison of the input–output responses of the rotary mechanism before and after optimization.

Input Values Δu/μm	Output Values Δθ/(″) Pre-Optimization	Input Values Δu/μm	Output Values Δθ/(″) Post-Optimization
1.01	1.01	1.06	1.86
1.67	1.85	1.65	2.91
2.34	2.96	2.37	4.25
3.21	4.03	3.36	6.10
3.85	4.90	3.91	7.12
4.51	6.14	4.46	8.15
5.37	7.18	5.42	9.94
6.14	7.83	6.19	11.37
6.84	8.84	6.91	12.71
7.43	9.55	7.38	13.59

**Table 4:** Comparison of the maximum output rotational displacement of this mechanism with other precision mechanisms.

Reference	Author	Year	Maximum Output Rotary Displacement (″)
[16]	Yang M. et al.	2021	9.56
This Paper	Na Zhang et al.	2025	15.34

**Table 5:** Comparison of the positioning error value of this mechanism with other precision mechanisms.

Reference	Author	Year	Positioning Error Value (″)
[14]	Yang, W.J. et al.	2021	2.98
[15]	Graser Philipp et al.	2021	1.3 (72.5 µrad)
[16]	Yang M. et al.	2021	0.85
[21]	Lorenzo, Iafolla et al.	2021	10
[36]	Liu, W.; Li, X. et al.	2018	7.2
This Paper	Na Zhang et al.	2025	0.67

## Data Availability

The data presented in this study are available from the corresponding author upon request.

## Abbreviations

The following abbreviations are used in this manuscript:

**Variable**	**Interpretation**	**Unit**
DOF	Degrees of freedom	\
PSU	Prismatic–spherical–universal	\
SPS	Spherical–prismatic–spherical	\
PSO	Particle swarm optimization	\
PZT	Piezoelectric actuator	\
a	Connecting element	\
b	Input motion element	\
c	Flexure hinge	\
d	Output motion element	\
$v_{i}^{k}$	The velocity of the ith particle at the kth iteration	\
$x_{i}^{k}$	The position of the ith particle at the kth iteration	\
$v_{i}^{k+1}$	The velocity of the ith particle at the (k + 1)th iteration	\
ω	The inertia weight factor	\
c_n_	The learning factor	\
rand_n_	A random number between 0 and 1	\
gbest	The best solution found by the population so far	\
pbest	The optimal solution currently found by the population’s fitness	\
x_n_	The x-coordinate of point n	mm
y_n_	The y-coordinate of point n	mm
R_n_	The distance from point n to the origin o	mm
θ_n_	The angle between the line connecting point n to the origin o and the X-axis	Angle (°)
l_ab_	The length of rod AB before the change in motion	mm
L_a′b′_	The length of rod AB after the change in motion	mm
Δv	The input displacement of the piezoelectric ceramic	μm
Δu	Half of the input displacement of the piezoelectric ceramic	μm
Δθ	The rotational angle generated by the actuator input on the micro-motion output mechanism d	Angle (″)
k_n_	Process variable	mm
${x_{1},x}_{2}{,x}_{3}$	The abscissa values of flexure hinges 1, 2, and 3	mm
$x_{4},x_{5}{,x}_{6}$	The ordinate values of flexure hinges 1, 2, and 3	mm
U	The driving voltage	V
δy_n_	The displacement variation of the side-mounted displacement sensor n along the Y-axis direction	μm
δx_n_	The displacement measured by the linear displacement sensor n in the X-axis direction	μm
Δθ_1max_, Δθ_2max_	The maximum output angular displacement during the ascending and descending phases of the optimized micro-motion rotary mechanism	Angle (″)
e_1max_, e_2max_	The maximum positioning error during the ascending and descending phases of the optimized micro-motion rotary mechanism	Angle (″)
